# Mobile applications for promoting and supporting breastfeeding: Systematic review and meta‐analysis

**DOI:** 10.1111/mcn.13733

**Published:** 2024-10-11

**Authors:** Monika Ziebart, Michael Kammermeier, Berthold Koletzko, Bernadeta Patro‐Golab

**Affiliations:** ^1^ Stiftung Kindergesundheit, c/o Dr. von Hauner Children's Hospital, LMU University Hospital Munich Germany; ^2^ Department of Pediatrics, Division of Metabolic and Nutritional Medicine Dr. von Hauner Children's Hospital, University Hospital, LMU Munich Munich Germany; ^3^ German Center for Child and Adolescent Health Munich Germany

**Keywords:** breastfeeding, infant nutrition, lactation, mHealth, mobile application, systematic review

## Abstract

Breastfeeding practices require improvement. We performed a systematic review of randomised controlled trials (RCTs) and analytic observational studies to assess effects of mobile applications (apps) aiming to support and promote breastfeeding targeting pregnant women, mothers of infants or their partners, on breastfeeding outcomes. We searched MEDLINE, EMBASE, Cochrane CENTRAL and Association of Computing Machinery Digital Library from 1 July 2008 to 29 November 2022, with lack of coverage of the most recent period before publication date being a limitation of this review. We performed meta‐analyses of findings from RCTs on primary outcomes, namely early breastfeeding initiation, exclusive and any breastfeeding rates. Joanna Briggs Institute tools were used for risk of bias assessment. Six RCTs, one quasi‐experimental and two cohort studies, mainly from high‐income countries, were included. Most studies focused on maternal app usage starting from pregnancy. One study targeted fathers as app‐users. Population characteristics, such as parity or delivery mode, apps scope of content and applied active components varied between studies. Main methodological limitations of studies were baseline differences between groups and lack of blinding. Compared to controls, app usage tended to increase the odds of exclusive breastfeeding. This nonsignificant effect was most pronounced at 1–1.5 months (*n* = 1294, odds ratio 1.45 (95% Confidence Interval, CI 0.83, 2.54), with considerable heterogeneity between studies [*I* ^2^ 77%]), but less so at 3 and 6 months post‐partum. The odds of early breastfeeding initiation, any breastfeeding at all time points were similar among groups. However, two cohort studies reported increased odds of exclusive and/or any breastfeeding at different time points. In conclusion, evidence is insufficient to show sustained beneficial effects of breastfeeding promotion and support through mobile apps on breastfeeding rates.

## Key messages

- Interventions aiming to promote and support breastfeeding through mobile apps are heterogeneous in terms of scope of content, active components used and mechanisms of action.
- Parental app usage tended to increase the odds of early exclusive breastfeeding, but no effects were observed on any breastfeeding.
- As development of digital interventions advances, more studies are needed to investigate the effects of digital tools on breastfeeding practices. These studies should take into account quality of such interventions and different clinical settings such as low‐ and middle‐income countries.

## Graphical abstract

In a meta‐analysis of randomised controlled trials, parental use of a mobile app promoting and supporting breastfeeding tended to increase the odds of early exclusive breastfeeding, but no effects were observed on any breastfeeding.

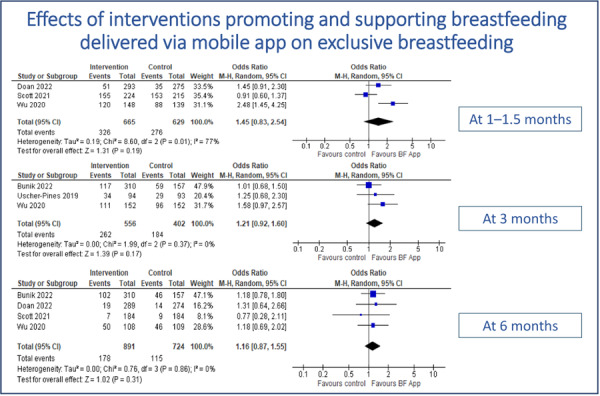

## 1 INTRODUCTION

Breastfeeding is considered the best feeding choice for infants associated with many short‐ and long‐term health benefits for mother and child (Pérez‐Escamilla et al., 2023). Protective effects of breastfeeding appear to be dose‐dependent, highlighting the role of exclusivity and duration of breastfeeding (Victora et al., 2016). The World Health Organisation (WHO) recommends exclusive breastfeeding for the first 6 months after birth and continued breastfeeding up to 2 years of age or beyond (WHO, 2023), but this is not achieved in the majority of infants worldwide, both in low‐ and middle‐income countries and in high‐income countries (Vaz et al., 2021). On an individual level, different digital health interventions may contribute to enhancing breastfeeding practices (Tomori et al., 2022). Interventions delivered with the use of digital technologies can utilise multiple active components and can be complex (Murray et al., 2016). Mobile applications (apps), that is, software programmes running on a mobile device (e.g., smartphone or tablet) (Maaß et al., 2022) offer ‘the possibility of personalising, tailoring, and adapting behaviour change interventions based on real‐time user and environmental data to improve health outcomes’ (Grundy, 2022). Many mobile health apps focusing on infant nutrition and targeting breastfeeding mothers and their partners have been developed by governmental, university‐affiliated, commercial and other providers and are available to parents (Cheng et al., 2020). While interventions promoting and supporting breastfeeding delivered via mobile apps were reported to be feasible and acceptable (Jaiswal et al., 2024; Uscher‐Pines et al., 2020; Wu et al., 2021), their effects on breastfeeding practices remain unclear. Systematic reviews and meta‐analyses assessing the effects of the use of mobile health or remote interventions in relation to breastfeeding outcomes pooled data from studies on wide range of interventions. These interventions included pamphlets, text messages, phone calls, as well as higher‐tech interventions as those delivered through mobile apps (Gavine et al., 2022; Qian et al., 2021). While these systematic reviews overall indicated some beneficial effects of the assessed interventions, considerable heterogeneity of the obtained findings limits their interpretation. Since new studies that assessed the effects of mobile apps on breastfeeding exclusivity and duration have provided inconsistent results (Vila‐Candel et al., 2024; Wu et al., 2020), we aimed to systematically assess the evidence on effects of mobile apps promoting and/or supporting breastfeeding on breastfeeding initiation, duration and exclusivity at different time points.

## 2 METHODS

This systematic review was performed according to the Cochrane guidelines (Higgins et al., 2023). The protocol of the review was registered at PROSPERO International prospective register of systematic reviews. Ethics approval was not required as the data analysed were in the public domain. PRISMA guidelines were used for reporting (Page et al., 2021).

### 2.1 Inclusion and exclusion criteria

We defined our inclusion and exclusion criteria following Population, Intervention, Comparison and Outcome framework.

#### 2.1.1 Population

We included studies that either enroled pregnant women and/or their partners, or mothers of infants up to 1 year of age and/or their partners/fathers. Studies that involved special population groups (e.g., mothers with underlying chronic diseases) or specific demographic subsets (low‐ or high‐income countries, certain ethnic groups) were also eligible for inclusion. Studies that targeted non‐family caregivers or those other than pregnant women/maternal partner, as well as health care professionals providing care to pregnant women/mothers of newborns or infants were excluded.

#### 2.1.2 Intervention/exposure

Studies that investigated pre‐ and/or post‐natal use of a mobile app providing any type of breastfeeding promotion (e.g., information on breastfeeding benefits or evidence‐based infant nutrition recommendations) or support (e.g., practical tips for lactating mothers or advice on managing common breastfeeding problems) were eligible for inclusion. Additionally, we considered eligible studies with mobile apps as a co‐intervention, if the mobile app was one of the main delivery media used for breastfeeding promotion or support. Application's design, quality, type of provider, content were not eligibility criteria. Similarly, we did not apply any restrictions to intensity or duration of the intervention/exposure. We excluded studies that assessed desktop or web applications or interventions that solely involved other technologies.

#### 2.1.3 Comparison

Eligible control group received setting and time specific standard care or a mobile app with no reference to breastfeeding. Studies with a different mobile app as a comparator that also promotes or supports breastfeeding were excluded.

#### 2.1.4 Outcomes

The primary outcomes of interest included early breastfeeding initiation, breastfeeding duration and exclusivity. We applied no restrictions to specific effect measures and considered for inclusion studies reporting observed effects as continuous measure (e.g., mean difference in duration of breastfeeding) or binary measure: relative and absolute effect (as reported by the authors of original studies). Only studies reporting on at least one of the primary outcomes were eligible. Our secondary outcomes included: breastfeeding intention, breastfeeding knowledge, maternal confidence and self‐efficacy, breastfeeding‐related complications and adverse effects (e.g., maternal anxiety, depression). We were also interested in usability of an app as reported by the users.

#### 2.1.5 Study design

Eligible study designs included randomised controlled trials (RCTs), quasi‐experimental studies and analytic observational studies with concurrent control group. Case reports/case series, before‐after studies, editorials, letters to the editor were excluded.

### 2.2 Types of sources

We searched four electronic databases: MEDLINE (Pubmed), EMBASE (OVID), Association of Computing Machinery Digital Library and Cochrane CENTRAL Register of Controlled Trials from 1 July 2008 to 8 August 021, with an update search ultimately covering records published up until 29 November 2022. We restricted the search timeframe to records published within the last 15 years due to the fact that the intervention of interest was not commonly available earlier. Additionally, we screened the *clinicaltrials.gov* registry and the reference lists of included studies and identified relevant review articles.

### 2.3 Search strategy

We developed our search strategy using a combination of breastfeeding‐related terms and mobile app‐related terms. Our search covered subject headings specific to each database (e.g., MeSH terms for MEDLINE) and free text words relevant to the research topic. Details on search strategy for each database are provided in the online Appendix S1. Articles published in languages other than English, German or Polish were not considered for inclusions due to feasibility reasons.

### 2.4 Selection of studies

Before screening, we combined records obtained from selected databases and removed duplicates using Sync Configuration of EndNote. Titles and abstracts yielded by the search were independently preliminarily screened against the inclusion criteria by two reviewers (B. P. G., M. Z., M. K.). After exclusion of clearly irrelevant studies, two reviewers (B. P. G., M. Z., M. K.) independently screened the full‐text articles against the inclusion criteria. Disagreements were resolved through discussion. Two authors were contacted for further information as it was not clear based on reported data if the identified records meet the inclusion criteria (Doan et al., 2022; Miremberg et al., 2022).

### 2.5 Data extraction

For data extraction we used evidence tables that were designed for this purpose. Data was extracted by two reviewers independently (M. K., M. Z.) and later cross‐checked by a third reviewer (B. P. G.). Any discrepancies were resolved by discussion. We contacted the authors of one study to obtain additional numerical data necessary for inclusion in our meta‐analysis (Bunik et al., 2022). The following items were extracted: study design, recruitment dates, country, study setting, sample size, characteristics of target population, intervention, comparison, outcomes measures, results and developer/provider of the app.

### 2.6 Data analysis and synthesis

For primary outcomes we performed quantitative analysis pooling results from included RCTs with the use of RevMan 5.4 software (Review Manager 5 RevMan 5, 2020). We applied a random effect model assuming that effect estimates can vary between included studies due to differences in the intervention effect in each individual study and sampling variability (Riley et al., 2011). We presented pooled effect estimates as odds ratio (OR) with 95% confidence interval (CI). For meta‐analysis, we used crude data (breastfeeding rates) as per intention‐to‐treat analysis that were provided by the authors of original studies. If the authors reported only proportion of breastfeeding women at a certain time point, together with the analysed sample size per study arms, we calculated the number of those breastfeeding to perform the meta‐analysis. Findings from non‐RTCs and on secondary outcomes were summarised narratively and in respective tables (quantitative pooling not possible due to limited number of contributing studies and/or variability in outcomes measures). Statistical heterogeneity between the studies was assessed based on *I* ^2^ statistic. We also visually inspected generated forest plots (overlap of CIs). We did not perform the assessment of publication bias by testing for funnel plot asymmetry due to the limited number of contributing studies (<10) in pooled analyses.

#### 2.6.1 Subgroup analysis

In case of one outcome, to explore high heterogeneity between the study findings, we performed a subgroup analysis based on app target users (mothers vs. fathers).

### 2.7 Assessment of risk of bias

Two reviewers (M. K., M. Z.) independently assessed the risk of bias using Joanna Briggs Institute critical appraisal tools (Barker et al., 2023). This assessment was later cross‐checked by a third reviewer (B. P. G.). Disagreements were resolved through discussion.

## 3 RESULTS

### 3.1 Study selection and characteristics

**Figure 1:**
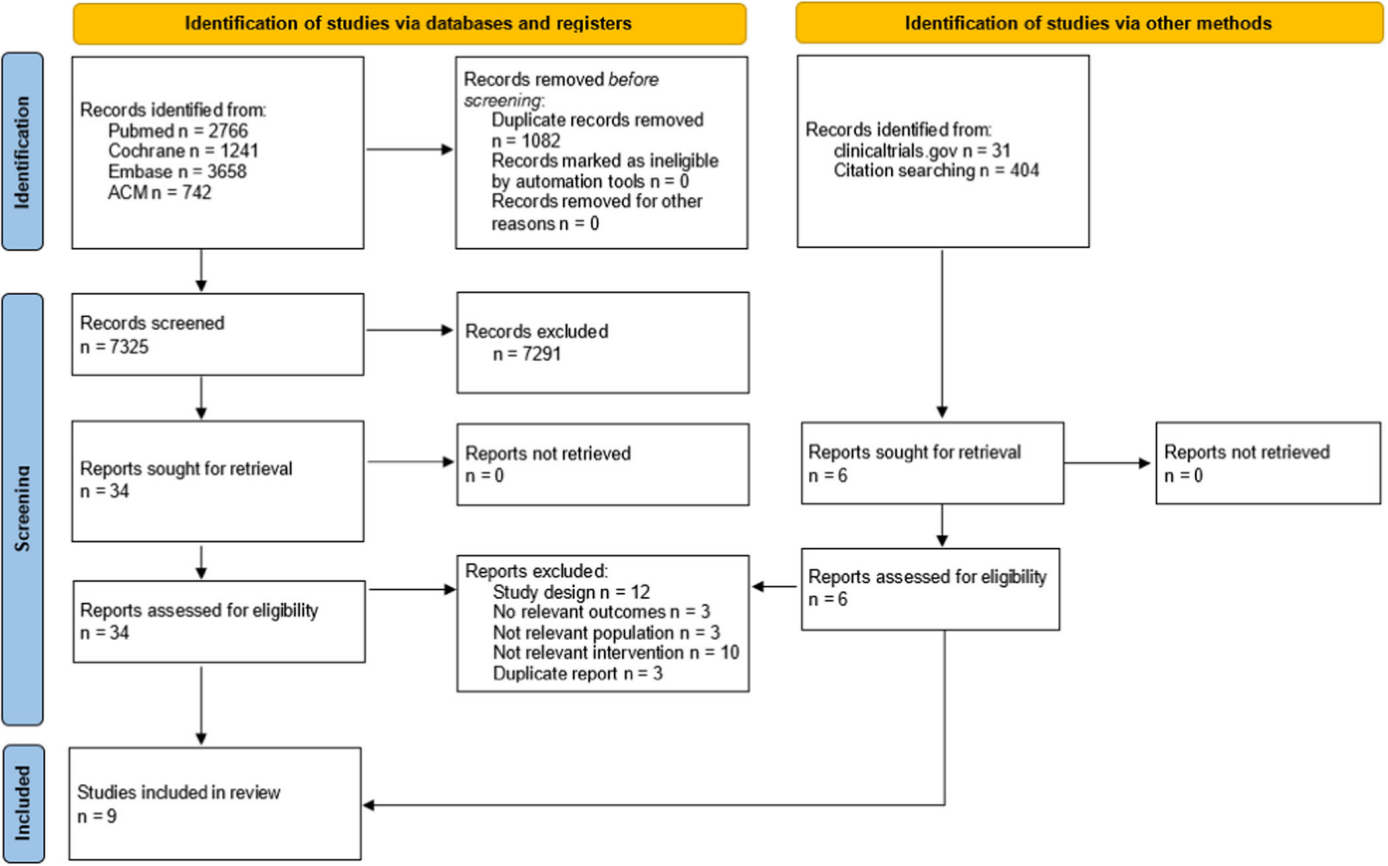
Preferred Reporting Items for Systematic Review and Meta‐Analysis (PRISMA) flow diagram detailing study selection process.

Out of 8636 records identified, nine studies (Borgen et al., 2019; Bunik et al., 2022; Cawley et al., 2020; Deave et al., 2019; Doan et al., 2022; Laws et al., 2018; Scott et al., 2021; Uscher‐Pines et al., 2020; Wu et al., 2020) were included in this systematic review. The details on the study selection process are shown in Figure 1. Excluded studies are listed in the online Appendix S2. Of included studies, six were RCTs (Borgen et al., 2019; Bunik et al., 2022; Doan et al., 2022; Scott et al., 2021; Uscher‐Pines et al., 2020; Wu et al., 2020), one was a quasi‐experimental study (Laws et al., 2018) and two were cohort studies (Cawley et al., 2020; Deave et al., 2019). Seven studies were conducted in countries with a very high Human Development Index (HDI) (The United States, Norway, England, Australia), and two in countries of a high HDI (China, Vietnam), with single studies recruited participants specifically in socioeconomically disadvantaged (Laws et al., 2018) or rural underserved communities (Uscher‐Pines et al., 2020). Five out of nine studies recruited pregnant women (Borgen et al., 2019; Bunik et al., 2022; Deave et al., 2019; Doan et al., 2022; Wu et al., 2020). Among those two studies enroled exclusively primiparous women (Bunik et al., 2022; Deave et al., 2019), one study focused on women with gestational diabetes mellitus (GDM) (Borgen et al., 2019), and one on those who delivered via caesarean section (Doan et al., 2022). In other studies, women were recruited after delivery (Cawley et al., 2020; Uscher‐Pines et al., 2020), or prenatally as well as post‐partum (Laws et al., 2018). Only one RCT enroled fathers‐to‐be as app‐users (Scott et al., 2021). Breastfeeding/infant feeding was a central theme of the app‐delivered intervention in five studies (Bunik et al., 2022; Doan et al., 2022; Scott et al., 2021; Uscher‐Pines et al., 2020; Wu et al., 2020). In other studies, interventions addressed broader themes, providing insights and guidance on healthy pregnancy, infant care and parenting (Cawley et al., 2020; Deave et al., 2019). Management of GDM (Borgen et al., 2019) or obesity prevention (Laws et al., 2018) were the overarching themes in other studies, with breastfeeding as one of the sub‐themes. Content‐wise these interventions mainly provided information on breastfeeding benefits and its importance, recommendations on infant feeding, support and practical advice on breastfeeding, or information regarding maternal diet and physical activity. In one RCT the app was used exclusively to provide a service, that is, on‐demand video calls with lactation consultants (Uscher‐Pines et al., 2020). Many of these interventions utilised a wide range of active components, features or functionalities, including videos, push notifications, ‘gaming’ elements, conversation forum or feeding trackers. In two studies, Facebook groups activity and emails with messages and links to website were used as a co‐intervention (Bunik et al., 2022; Laws et al., 2018). The timing and duration of the app usage varied covering mainly the period from pregnancy up to several months post‐partum. Only two studies introduced the app after the child was born (Laws et al., 2018; Uscher‐Pines et al., 2020). In most studies the designs of the apps were underpinned by different theories mainly by Social Cognitive Theory, though some studies did not refer to any theoretical framework (Cawley et al., 2020; Wu et al., 2020) (Table 1). The control groups in all studies were provided with the usual care, while in one RCT (Bunik et al., 2022) injury prevention texts and in another RCT (Doan et al., 2022) general maternal and child care messages were provided. Maternal self‐reported data on breastfeeding practices was collected mainly through online questionnaires or through survey completed by phone. Primary outcomes of our interest reported in individual studies included early breastfeeding initiation, rates of exclusive and any breastfeeding at different time points, and breastfeeding duration. Follow‐up period varied, ranging from three to 9 months post‐partum. Details on all study characteristics are presented in Table 1.

**Table 1:** Characteristics of included studies.

Study ID	Study design; recruitment dates	Country, study setting, sample size,	Target app users	Exposure/intervention Content and features/functions of the app: Theoretical framework applied Co‐intervention, if any	Timing/duration of the intervention	Comparison	Comments
Borgen et al. (2019)	RCT October 2015–April 2017	Norway; diabetes outpatient clinics; *N* = 238/233	Pregnant women with GDM, <33 weeks of gestation at recruitment	Pregnant + smartphone mobile app + usual care The app primarily designed to support the management of GDM, but provided information on the advantages of BF (e.g., maintaining stable blood glucose in the new‐born baby) + FAQ (e.g., ‘Can I breastfeed?’) Theoretical framework: Health Belief Model	Prenatal period	Usual care	BF as secondary outcome; power calculation done for primary (glycaemic) outcomes
Bunik et al. (2022)	RCT September 2018–January 2019	USA, national sample, recruited online *N* = 469/346	Primiparous singleton pregnant women ≥36 weeks of gestation at recruitment	Mothers Milk Messaging mobile app Prenatally text messages on increasing perceived benefits, attitudes, positive outcome expectancies and BF related self‐efficacy; post‐partum messages on strategies to garner social support/enhance behavioural skills and self‐efficacy to overcome BF barriers; videos imbedded on the app and linked through YouTube; a digital story workshop with Story Centre. Content from the BF Telephone Triage and Advice book in a scrolling format by topic; feeding tracker; Theoretical framework: social cognitive theory and the theory of planned behaviour Co‐intervention: text messages ± physician moderated private Facebook (groups combined due to minimal Facebook activity)	3–4 weeks before delivery and up to 3 months post‐partum	Attention control: injury prevention texts	‐
Cawley et al. (2020)	Retrospective cohort study March 2018–January 2019	USA, selected providence clinics *N* = 567/541	Pregnant women who at recruitment gave birth to an infant in the past 4–6 months	Circle by Providence mobile app Personalised, evidence‐based health information on pregnancy, post‐partum recovery and infant care, including BF support in the form of videos, articles and a guide to local resources, to‐do lists, reminders related to prenatal and paediatric care, including tools to track feeding Theoretical framework: NA	Prenatal period	App nonusers	As of September 2018, the app available to patients across all Providence St. Joseph Health locations
Deave et al. (2019)	Cohort study September 2016–February 2017	England, maternity units from five geographical sites *N* = 488/296	Pregnant women, in first pregnancy; 12–16 weeks of gestation at recruitment	Pregnancy and parenting Baby Buddy mobile app + usual care Evidence‐based interactive information, support, guidance, empowerment for pregnancy and first 6 months of child's life, including BF promotion and support: a user‐designed interactive avatar as a ‘gaming’ element; videos; engaging and interactive daily information to support healthy behaviours including BF Theoretical framework: self‐efficacy theory	Any use during pregnancy up to 3 month post‐partum (study period)	No Baby buddy app downloaded	BF outcomes—secondary ones analysed post hoc
Doan et al. (2022)	RCT May 2020–July 2021	Vietnam, urban hospitals *N* = 1266/563 (lost to FU 156, but only 563 delivered by CS)	Mothers with singleton pregnancy, who delivered by CS; 24–36 weeks of gestation at recruitment	Mobile app with BF content Information, including visual aids, encouraging exclusive BF; text notifications with behaviour change messages and suggestions for further resources in the app's library. Prenatally the focus on: BF importance, benefits, ability of mothers to initiate BF early and to exclusively BF regardless of mode of delivery after birth: messages on the importance of exclusive BF, addressing common BF problems, maternal diet/physical activity etc. BF content based on the national and international guidelines Theoretical framework: social cognitive theory, COM‐B system	The app auto‐generated notifications during pregnancy and after delivery for 6 months (trice and twice weekly, respectively)	Messages on general maternal and child health care, sent via app	Study during Covid‐19—lockdown periods, with conflicting information on BF in social media; differences in the length of exposure to the intervention
Laws et al. (2018)	Quasi‐experimental study NA	Australia; participants from socioeconomically disadvantaged communities *N* = 645/518	Pregnant women, >30 weeks of gestation or parent/carer of an infant <3 month; Average age at enrolment 7–8 weeks post‐partum	mHealth obesity prevention intervention (Growing Healthy programme) accessed via mobile app and/or website/SMS; Evidence‐based article and videos with practical advice and strategies consistent with national guidelines on infant feeding, including promotion of BF. Additionally, information on infant sleep, settling and general support for parents provided; app‐generated notifications to age‐ and feeding‐specific app content sent Theoretical framework: Behaviour Change Wheel and the Capability, Opportunity and Motivation model of behaviour change Co‐intervention: emails with messages and links to website (similar content as the app); Facebook group with messages posted by the moderator	From <3 month up to 9 month of age	Usual care	Feasibility study, not powered to detect differences in BF rates; high rates of exclusive BF at baseline
Scott et al. (2021)	RCT August 2015–December 2016	Australia, public and private hospitals, *N* = 1426/702 couples	Fathers‐to‐be who intended to co‐participate in child rearing	Milk Man, BF mobile smartphone app (one of the four study arms, *n* = 397) Gamification, conversation forum and push notifications (2/week) linking to polls and conversation starters to engage fathers with BF information; parenting information and links to external websites/BF support services also provided Theoretical framework: social cognitive theory	Approximately 32 weeks of pregnancy up to 6 months post‐partum	Usual care	Sample not representative of the general population of expecting parents due to self‐selection (socially advantaged, highly educated sample)
Uscher‐Pines et al. (2019)	RCT October 2016–May 2018	USA, rural county hospital (Health Professional Shortage Area), *N* = 203/187	Mothers with singleton baby (≥35 weeks of gestational age), who initiated BF	Pacify Health's telelactation mobile app Free, unlimited on‐demand video calls with lactation consultants (IBCLCs) Theoretical framework: individualised support to improve BF self‐efficacy	After delivery until 12 weeks post‐partum	Usual care	The study designed to assess feasibility of telelactation, not powered to detect differences in BF rates
Wu et al. (2020)	RCT May 2019–April 2020	China, hospitals in 13 townships in Huzhu County, *N* = 344/217	Singleton, pregnant women, 11–37 weeks of gestation at recruitment	Special module ‘Optimal Feeding’ on the official account of the WeChat mobile phone app (an app‐within‐an‐app platform) Time adjusted/tailored and evidence‐based feeding messages, designed for BF promotion and education, that provided key BF knowledge and relevant infant feeding advice, addressing BF problems and preparation for BF and complementary feeding; In addition: a feeding knowledge competition, a baby growth chart and an online forum provided Theoretical framework: NA	From 3 months of pregnancy up to 6 months post‐partum	Usual care/no access to the module	Possible risk of contamination between the groups within the same township

Abbreviations: App, application; BF, breastfeeding; COM‐B, capability, opportunity, motivation, and behaviour; CS, caesarean section; FAQ, frequently asked questions; FU, follow‐up; GDM, gestational diabetes mellitus; IBCLC, International Board Certified Lactation Consultant; ITT analysis, intention‐to‐treat analysis; N, number; NA, not available; NHS, National Health Service; RCT, randomised controlled trail.

### 3.2 Risk of bias assessment

Assessment of the risk of bias for each individual study is provided in the online Appendix S3. Although true randomisation was used to assign participants to the study groups in all RCTs, allocation concealment was unclear (Bunik et al., 2022; Doan et al., 2022) or not achieved (Scott et al., 2021) in half of the reports. In four of six RCTs (Borgen et al., 2019; Bunik et al., 2022; Uscher‐Pines et al., 2020; Wu et al., 2020) and in a single quasi‐experimental trial (Laws et al., 2018), study groups differed at baseline in terms of characteristics potentially relevant for the intervention effect, including parity, type of health insurance or exclusive breastfeeding rates. Due to the nature of the intervention, blinding of the study participants, who also acted as outcome assessors in relation to self‐reported breastfeeding, could not be achieved. Reports from included RCTs were unclear to us with respect to received (if at all) breastfeeding support other than the study intervention as part of a usual/standard care, and blinding of those delivering this service in relation to study intervention. Baseline differences between the study groups (Cawley et al., 2020) and high rates of loss to follow‐up (Deave et al., 2019) were major limitations in the assessed cohort studies. We considered maternal self‐reports on breastfeeding practices provided in infancy as both valid and reliable (Li et al., 2005) in all studies. However, we acknowledge that these reports, when concerning exclusive breastfeeding, may be less accurate. Half of the assessed RCTs (Borgen et al., 2019; Uscher‐Pines et al., 2020; Wu et al., 2020) were underpowered to detect significant differences in breastfeeding outcomes between the study groups.

### 3.3 Primary outcomes

#### 3.3.1 Early initiation of breastfeeding

**Figure 2:**
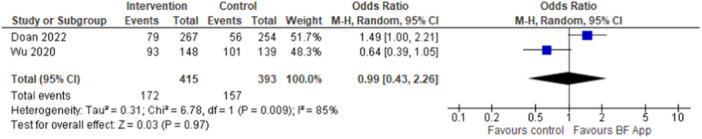
Forest plots of the effects of interventions promoting and supporting breastfeeding delivered via mobile app on early initiation of breastfeeding.

Early initiation of breastfeeding was assessed in two RCTs and was defined as an initiation of breastfeeding within one (Wu et al., 2020) or 2 h after birth (Doan et al., 2022). The pooled estimate indicated no difference between the app‐users and control group (OR 0.99; 95% CI [0.43, 2.26]) (Figure 2). We observed high statistical heterogeneity (*I* ^2^ = 85%) between the studies. Due to low number of available studies, we could not formally perform a subgroup or sensitivity analysis to explore this further. However, it seems likely that differences in study population characteristics (singleton, pregnant women vs. mothers who delivered specifically via caesarean section) might have contributed to the observed heterogeneity.

#### 3.3.2 Exclusive breastfeeding

**Figure 3:**
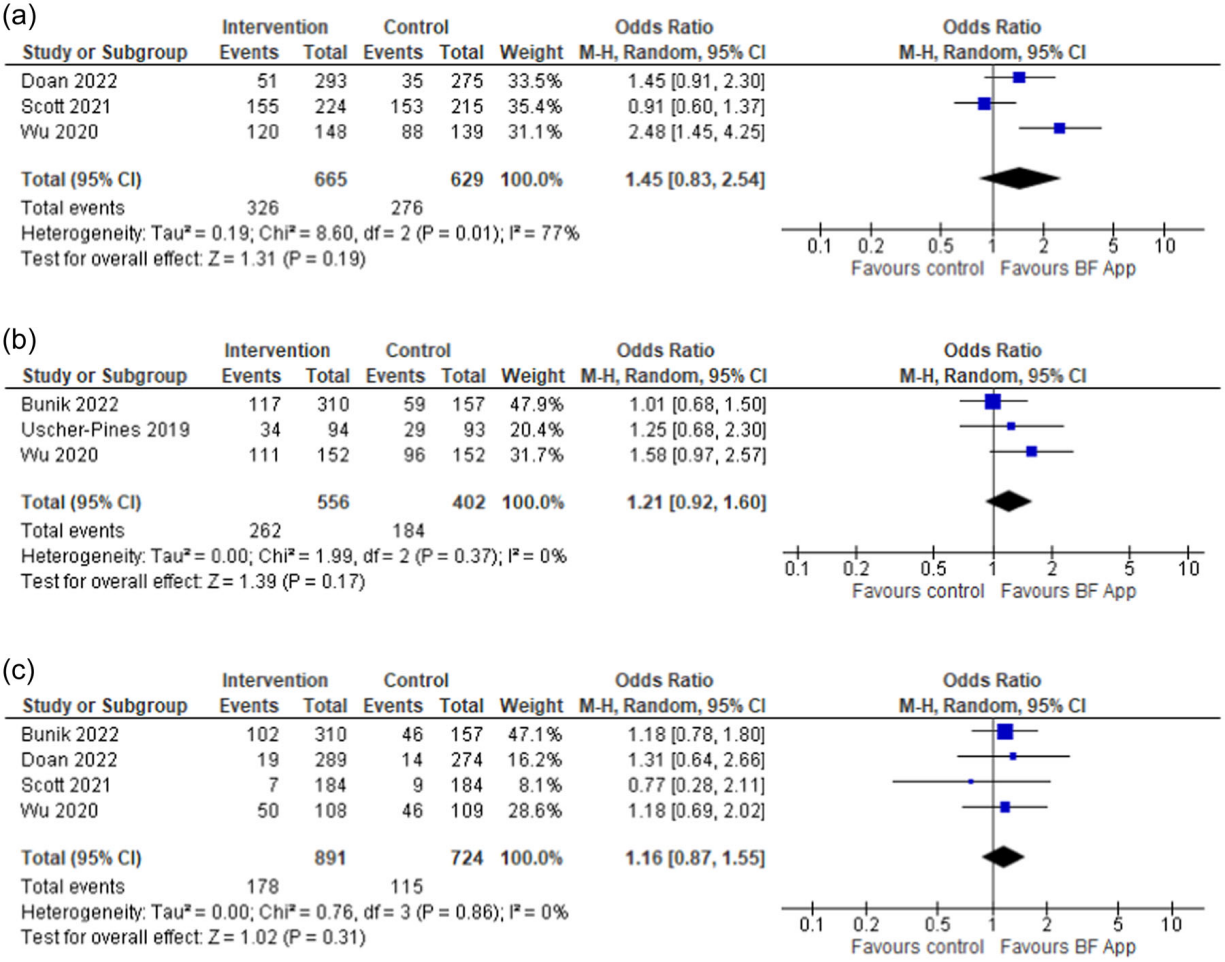
(a–c) Forest plots of the effects of interventions promoting and supporting breastfeeding delivered via mobile app on exclusive breastfeeding odds at (a) 1–1.5 months, (b) 3 months and (c) 6 months.

Rates of parental reported exclusive breastfeeding (or cessation of exclusive breastfeeding) were indicated by authors on individual studies at different time points, starting from the first week of life until the age of 6 months (Appendix S4). We pooled available results from included RCTs and performed meta‐analysis to assess the odds of exclusive breastfeeding at 1–1.5 months (*n *= 3) (Doan et al., 2022; Scott et al., 2021; Wu et al., 2020), at 3 months (*n *= 3) (Bunik et al., 2022; Uscher‐Pines et al., 2020; Wu et al., 2020) and at 6 months (*n *= 4) (Bunik et al., 2022; Doan et al., 2022; Scott et al., 2021; Wu et al., 2020) post‐partum. We noted a non‐significantly higher odds of exclusive breastfeeding at 1–1.5 months in the app group as compared to control (OR = 1.45; 95% CI [0.83, 2.54]) (Figure 3a) which attenuated further at 3 months (Figure 3b) and at 6 months post‐partum (Figure 3c). We observed considerable statistical heterogeneity of the included studies at 1–1.5 months (*I*² 77%), indicating that no generalisable conclusions can be drawn, but not at the other time points (*I*² 0% at 3 and at 6 months). A subgroup analysis based on app target users (mothers vs. fathers), as a priori planned in the review protocol, partly explained the variability between the study findings (*I*² 55%), showing a statistically significant effect on exclusive breastfeeding at 1–1.5 months with mothers enroled as target app users (two RCTs, OR = 1.86; 95% CI [1.10, 3.16]). A single quasi‐experimental study (Laws et al., 2018) showed no significant differences in the odds of exclusive breastfeeding at 6 months (Appendix S4). In a post hoc analysis from one prospective cohort study (Deave et al., 2019) maternal app usage was associated with non‐significantly greater odds of exclusive breastfeeding at 1 week and 1 month post‐partum (Appendix S4), and a statistically significant difference at 3 months (adjusted OR 1.79; 95% CI [1.02, 3.16]).

#### 3.3.3 Any breastfeeding

**Figure 4:**
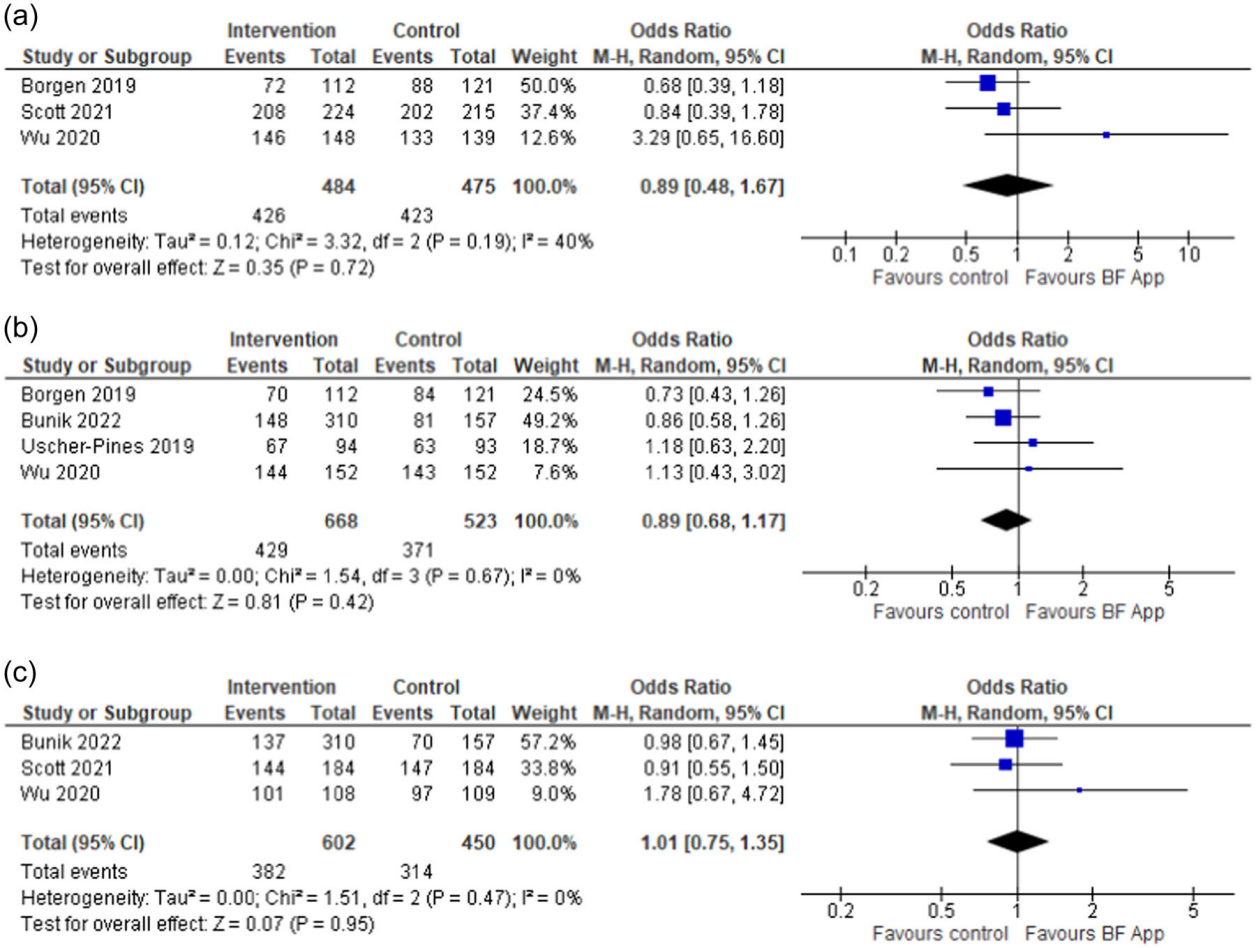
(a–c) Forest plots of the effects of interventions promoting and supporting breastfeeding delivered via mobile app on any breastfeeding rates at (a) 1–1.5 months, (b) 3 months and (c) 6 months.

We further compared the rates of any breastfeeding between the study groups reported in the included RCTs. At 1–1.5 months (*n *= 3), the odds of any breastfeeding were similar among app‐users and non‐users (OR = 0.89; 95% CI [0.48, 1.67])) (Borgen et al., 2019; Scott et al., 2021; Wu et al., 2020). Similarly, at 3 months (*n *= 4) (Borgen et al., 2019; Bunik et al., 2022; Uscher‐Pines et al., 2020; Wu et al., 2020) and 6 months after delivery (*n *= 3) (Bunik et al., 2022; Scott et al., 2021; Wu et al., 2020) there was no difference between study groups (OR = 0.89; 95% CI [0.68, 1.17]) and OR = 1.01 (95% CI [0.75, 1.35]), respectively. We did not observe considerable statistical heterogeneity between the study findings at any time point (Figure 4a–c). Likewise, there were no differences in breastfeeding rates and duration assessed at 9 months in one quasi‐experimental study (Laws et al., 2018) (Appendix S4). However, two cohort studies reported mothers using an app to be more likely to breastfeed their babies (Appendix S4). In one of these studies (Deave et al., 2019) the outcomes were assessed at 1 week, and 1 and 3 months, with the adjusted odds of any breastfeeding of 1.72 (95% CI [0.99, 2.99]) at 3 months. The other cohort study (Cawley et al., 2020) reported significantly higher odds of any breastfeeding at ≥6 months (adjusted OR 1.75, *p* = 0.01).

### 3.4 Secondary outcomes

Secondary outcomes within the scope of this review, assessed in individual studies with the use of different measures included: adverse events (*n *= 2), breastfeeding knowledge gain (*n *= 2) and self‐efficacy (*n *= 2), perceived partner/breastfeeding support (*n *= 2) and satisfaction with breastfeeding experience (*n *= 1) (Appendix S5). Apart from significantly higher breastfeeding self‐efficacy observed among app users in one study (Bunik et al., 2022), no significant differences between study groups were reported for other outcomes. In addition, no harms associated with the app use were observed (Borgen et al., 2019; Bunik et al., 2022).

### 3.5 App usage

Satisfaction with the app use was assessed in three studies (Bunik et al., 2022; Laws et al., 2018; Uscher‐Pines et al., 2020) and was reported as high. Analysis of various app usage metrics was conducted in six studies (Appendix S5) (Bunik et al., 2022; Deave et al., 2019; Laws et al., 2018; Scott et al., 2021; Uscher‐Pines et al., 2020; Wu et al., 2020). The proportion of participants from the intervention group who downloaded or registered the app reported in three studies ranged from 74.8% to >80% (Bunik et al., 2022; Laws et al., 2018; Scott et al., 2021). In one RCT where the app was used exclusively to provide a service, that is, on‐demand video calls with lactation consultants, 50% of the participants from the intervention group took part in one or more video calls (Uscher‐Pines et al., 2020). The level of engagement with the app was not associated with breastfeeding outcomes (Scott et al., 2021) or had a positive effect on breastfeeding, but no differential effect of engagement between group using intervention app versus control app was observed (Bunik et al., 2022).

## 4 DISCUSSION

We systematically assessed the evidence on effects of interventions with mobile apps promoting and supporting breastfeeding on breastfeeding outcomes. Although mothers who used the app tended to be more likely to exclusively breastfeed their babies in the early post‐partum period, no significant appreciable effect was observed at 3 and 6 months after delivery. App usage had no effect on the odds of any breastfeeding at any time point based on the evidence from RCTs.

Our findings are in line with a recent systematic review investigating remote provision of breastfeeding support and education, including via mobile apps (Gavine et al., 2022). This review of 29 RCTs showed no beneficial effects of these interventions on any breastfeeding. Although the authors reported a 25% risk reduction for cessation of exclusive breastfeeding at 3 months (low certainty in evidence), they did not observe any significant differences in the rates of exclusive breastfeeding between the study groups at other time points (4–8 weeks, at 6 months). The authors emphasised substantial heterogeneity of interventions included in this systematic review, defined as those where service users and service providers were separated by distance (e.g., text messaging, telephone, social media or video calls). This heterogeneity of interventions is likely to be associated with considerable heterogeneity of findings observed in performed meta‐analyses. Of note, the authors included only one RCT that was among included studies in our review, which can be explained by the differences in the timeframe of the search and in applied inclusion/exclusion criteria. Thus, we consider our review as an important addition to the existing evidence summaries.

Other recently published systematic reviews aimed to assess the effect of certain intervention groups, defined based on the common mode of their delivery or technology used to deliver these interventions (i.e., ‘mobile Health interventions’ [Qian et al., 2021], ‘internet‐based electronic technology interventions’ [Almohanna et al., 2020], ‘virtual lactation support’ [Blackmore et al., 2022]), on breastfeeding outcomes. Due to broadly defined intervention of interest, the scope of these reviews was much broader than that of our review, and different study selection criteria were applied. This, together with various time frames of the search applied, resulted in a very little (none or single studies) overlap between these reviews and our review in terms of included studies. Overall, these systematic reviews and meta‐analyses suggested beneficial effects on breastfeeding exclusivity; however, high heterogeneity observed in performed meta‐analyses raises concerns with regard to obtained results. We applied more narrow criteria to define interventions of interest, namely those delivered via mobile app that by definition are complex interventions. Analysing the characteristics of the mobile apps used in the studies included in this review, we consider that these interventions still may vary significantly in scope of content, active components used and mechanisms of action, all relevant for their effects. Following the principles of meta‐analysis conduct (Higgins et al., 2023), we suggest that a shift towards pooling the effects of less diverse interventions would benefit future meta‐analyses in providing accurate effect estimates. Since completion of our search, findings from recent RCTs conducted in countries of very high HDI and investigating the effects of interventions delivered via mobile app on breastfeeding outcomes have been published (Acar & Şahin, 2024; Saucedo Baza et al., 2023; Sevda & Sevil, 2023; Vila‐Candel et al., 2024). These RCTs, often small and with short follow‐up periods, showed higher rates of exclusive breastfeeding among app‐users, mainly in the first 2 months post‐partum. Two studies with 6‐month follow‐up found no difference in any breastfeeding rates between the study groups at this time point (Sevda & Sevil, 2023; Vila‐Candel et al., 2024). Thus, these studies tend to be in line with the findings of this systematic review.

We can only speculate as to why breastfeeding promotion and support delivered via mobile app showed no effects on any breastfeeding but tended to enhance the rates of early exclusive breastfeeding. There are many barriers that are commonly shared with regard to both exclusivity and duration of breastfeeding. While the awareness of the overall importance of breastfeeding seems to be widespread among mothers at least in high‐income countries, breastfeeding exclusivity is less appreciated as important among women who otherwise are determined to breastfeed their offspring (Cascone et al., 2019). Thus, knowledge gain alone (rather than behaviour change), specifically in the area of breastfeeding exclusivity, enabled through educational content commonly provided in mobile apps, may play a role in achieving desired outcomes (Skouteris et al., 2017). On the other hand, the lack of a significant and sustained impact of mobile app usage with respect to both breastfeeding exclusivity and duration may be due limitations in the rapidly developing technology‐enabled interventions having not yet reached their peak potential in supporting multiple aims (e.g., not only education, but also behaviour change support, monitoring functions and more), suggesting possible greater future potential (Murray et al., 2016). Further, the effectiveness of breastfeeding interventions in studies might differ depending on the extent of support provided to controls. Although ineffective in countries with high‐quality standard health care, the same interventions may prove beneficial in a more underserved setting. Therefore, we support the suggestions of a previous review (Tomori et al., 2022) that more studies are needed in low‐ and middle‐income countries, as well as in disadvantaged populations in all countries. Finally, it is important to note that digital interventions alone, acting on the individual level might not be sufficient to overcome breastfeeding barriers. As suggested in a recent Lancet series, more multi‐level and multi‐component interventions appear to be needed to improve breastfeeding practices (Pérez‐Escamilla et al., 2023).

### 4.1 Strengths and limitations

To our knowledge this is the first systematic review specifically addressing the role of mobile apps in improving breastfeeding practices. For its conduct we followed an a priori developed review protocol, publicly available online to ensure transparency. A comprehensive search strategy utilising several data sources, rigorous procedures on data selection process, data extraction and synthesis with the involvement of two independent reviewers, are among the strengths of this review. However, several limitations of this review need to be acknowledged too. The timeframe of our search did not cover the most recent period before publication date of this systematic review. Given how rapidly digital interventions have been developing recently, we can expect numerous new studies in this area in the near future. Therefore, further monitoring of the evidence should be encouraged. Included RCTs suffered from some methodological limitations, mainly lack of blinding, baseline differences between study groups and unclear allocation concealment. At the inception stage, the decision to additionally include observational studies in a review on the effectiveness of an intervention derived from the assumption that the number of eligible RCTs may be very limited. Yet, we acknowledge design‐related limitations of these studies, such as residual confounding. Studies included in our review were mainly conducted in high‐income countries, assuming that mothers in the control group did receive some kind of breastfeeding education and support as part of their routine prenatal and post‐natal care. However, in general the effect of breastfeeding promotion on breastfeeding rates was shown to be greater in developing than in developed countries (Imdad et al., 2011; Olufunlayo et al., 2019). To avoid considerable heterogeneity, we excluded studies with health care professionals as app users. Still our eligibility criteria allowed for inclusion of studies with both parents (mothers and fathers) as app target users, as well as of pregnant women/breastfeeding mothers with different clinical characteristics such as healthy pregnant women and those with pregnancy complications (i.e., GDM). A subgroup analysis based on app target users (mothers vs. fathers) partly explained the variability between the study findings on exclusive breastfeeding at 1–1.5 months. Thus, less broad inclusion criteria in relation to population of interest may be considered by the authors of future systematic reviews in this area. As we identified only one RCT involving fathers as mobile app users, more studies focused on this target group are needed to conclude if interventions delivered via mobile app aimed at a co‐parent can indeed improve breastfeeding outcomes. Similarly, more studies that focus on specific target groups among pregnant women and mothers, owning characteristics potentially influencing the intervention effects, would assist in better defining those who are likely to benefit the most from breastfeeding support provided through the use of mobile app. In our review, the number of RCTs enroling exclusively primiparous mothers, those who delivered via caesarean section, or those from disadvantaged area was limited, and so was the overall number of studies available for meta‐analysis at certain time points. While we extracted various data to best characterise tested interventions (e.g., theoretical framework, scope of content, app features/functionalities and active components used), we did not formally evaluate the overall quality of interventions used in individual studies, which may have impact on the assessed outcomes. Additionally, data regarding app use and non‐use by the study participants were reported inconsistently and only in some studies. Better reporting of app usage metrics across the studies would allow for more accurate interpretation of study findings.

## 5 CONCLUSION

Mobile apps may have great potential as a medium for interventions to promote and support breastfeeding, but currently available evidence is insufficient to support their effectiveness in improving breastfeeding practices. Further work to assess the impact of refined mobile app interventions, that should also target disadvantaged populations, is warranted.

## AUTHOR CONTRIBUTIONS

Bernadeta Patro‐Golab, Monika Ziebart and Berthold Koletzko conceptualised the review. Monika Ziebart, Michael Kammermeier and Bernadeta Patro‐Golab performed data search and study selection, data extraction and analysis. Monika Ziebart, Bernadeta Patro‐Golab and Michael Kammermeier prepared the draft manuscript. All authors reviewed and revised the manuscript and approved the final version.

## CONFLICT OF INTEREST STATEMENT

The authors declare no conflict of interest.

## Supporting information

Supporting information.

## Data Availability

As this publication is a systematic review, the data that support the findings are articles published in academic journals that are already in the public domain. Therefore, data sharing is not applicable to this article as no new data were created or analysed.
