# Supplementary material for: Mobile applications for promoting and supporting breastfeeding: Systematic review and meta‐analysis
Source: Matern Child Nutr. 2024 Oct 11;21(1):e13733. doi: 10.1111/mcn.13733 (PMC11650046; doi:10.1111/mcn.13733)
Supplement: Supplementary file 1 — Supporting information. [file MCN-21-e13733-s001.pdf]

SUPPLEMENTARY MATERIALS

Mobile applications for promoting and supporting breastfeeding: systematic review and meta-analysis

Appendices:

Appendix 1. Detailed search strategy
Appendix 2. Excluded studies and reasons for exclusion
Appendix 3. Assessment of the risk of bias in individual studies A. RCTs, B. quasi-experimental studies, C. cohort studies
Appendix 4. Primary outcomes measures and results from individual studies
Appendix 5. Secondary outcomes results

Appendix 1. Detailed search strategy

PubMed, searched 29/11/2022

1. "Breast Feeding"[MeSH Terms]
2. "Child Nutrition Sciences"[MeSH Terms]
3. "Infant Nutritional Physiological Phenomena"[MeSH Terms]
4. "Feeding Behavior"[MeSH Terms]
5. "infant care"[MeSH Terms]
6. "perinatal care"[MeSH Terms]
7. "postnatal care"[MeSH Terms]
8. "infant health"[MeSH Terms]
9. "parenting"[MeSH Terms]
10. "breastfeeding"[Text Word]
11. "breastfed"[Text Word]
12. "breast Feeding"[Text Word]
13. "breast fed"[Text Word]
14. "perinatal health"[Text Word]
15. "postnatal health"[Text Word]
16. "perinatal care"[Text Word]
17. "postnatal care"[Text Word]
18. "feeding"[Text Word]
19. "infant nutrition*"[Text Word]
20. "lactation"[Text Word]
21. "lactating"[Text Word]
22. "breast milk"[Text Word]
23. "infant care"[Text Word]
24. "child nutrition*"[Text Word]
25. "Feeding Behavior"[Text Word]
26. "infant health"[Text Word]
27. "parenting"[Text Word]
28. "Mobile Applications"[MeSH Terms]
29. "Smartphone"[MeSH Terms]
30. "Cell Phone"[MeSH Terms]
31. "computers, handheld"[MeSH Terms]
32. "Telemedicine"[MeSH Terms]
33. "mobile phone"[Text Word]
34. "cell phone"[Text Word]
35. "smartphone"[Text Word]
36. "IOS"[Text Word]
37. "iPhone"[Text Word]
38. "Android"[Text Word]
39. "iPad"[Text Word]
40. "handheld computer"[Text Word]
41. "personal digital assistant"[Text Word]
42. "app"[Text Word]
43. "apps"[Text Word]
44. "mobile app"[Text Word]
45. "mobile application"[Text Word]
46. "mobile apps"[Text Word]
47. "mobile health"[Text Word]
48. "mHealth"[Text Word]
49. "m-health"[Text Word]
50. "electronic health"[Text Word]
51. "eHealth"[Text Word]
52. "e-health"[Text Word]
53. "digital health"[Text Word]
54. "telehealth"[Text Word]
55. "Telemedicine"[Text Word]
56. "telelactation"[Text Word]
57. "mobile technolog*"[Text Word]
58. "portable software"[Text Word]

59. "portable"[Text Word]
60. "portable electronic"[Text Word]
61. "wearable device"[Text Word]
62. "wireless"[Text Word]
63. 1 or 2 or 3 or 4 or 5 or 6 or 7 or 8 or 9 or 10 or 11 or 12 or 13 or 14 or 15 or 16 or 17 or 18 or 19 or 20 or 21 or 22 or 23 or 24 or 25 or 26 or 27
64. 28 or 29 or 30 or 31 or 32 or 33 or 34 or 35 or 36 or 37 or 38 or 39 or 40 or 41 or 42 or 43 or 44 or 45 or 46 or 47 or 48 or 48 or 49 or 50 or 51 or 52 or 53 or 54 or 55 or 56 or 57 or 58 or 59 or 60 or 61 or 62
65. 63 and 64
66. limit 65 to yr="2008 -2022"

Embase searched via OVID on 29/11/2022

1. Breastfeeding.tw.
2. Breastfed.tw.
3. breast feeding.tw.
4. breast fed.tw.
5. perinatal health.tw.
6. postnatal health.tw.
7. infant health.tw.
8. perinatal care.tw.
9. postnatal care.tw.
10. infant care.tw.
11. feeding.tw.
12. infant nutrition*.tw.
13. lactation.tw.
14. lactating.tw.
15. breast milk.tw.
16. child nutrition*.tw.
17. feeding behavior.tw.
18. child parent relation.tw.
19. breast feeding.sh.
20. nutritional sciences.sh.
21. infant nutrition.sh.
22. feeding behavior.sh.
23. infant care.sh.
24. perinatal care.sh.
25. postnatal care.sh.
26. child health.sh.
27. child parent relation.sh.
28. mobile phone.tw.
29. cell phone.tw.
30. smartphone.tw.
31. IOS.tw.
32. iPhone.tw.
33. Android.tw.
34. iPad.tw.
35. handheld computer.tw.
36. personal digital assistant.tw.
37. app.tw.
38. apps.tw.
39. mobile app.tw.
40. mobile apps.tw.
41. mobile application.tw.
42. mobile health.tw.
43. mHealth.tw.
44. m-Health.tw.
45. electronic health.tw.
46. eHealth.tw.
47. e-Health.tw.
48. digital health.tw.
49. telehealth.tw.
50. telemedicine.tw.

51. telelactation.tw.
52. mobile technolog*.tw.
53. portable software.tw.
54. portable.tw.
55. portable electronic.tw.
56. wearable device OR wireless.tw.
57. mobile application.sh.
58. smartphone.sh.
59. mobile phone.sh.
60. personal digital assistant.sh.
61. telemedicine.sh.
62. 1 or 2 or 3 or 4 or 5 or 6 or 7 or 8 or 9 or 10 or 11 or 12 or 13 or 14 or 15 or 16 or 17 or 18 or 19 or 20 or 21 or 22 or 23 or 24 or 25 or 26 or 27
63. 28 or 29 or 30 or 31 or 32 or 33 or 34 or 35 or 36 or 37 or 38 or 39 or 40 or 41 or 42 or 43 or 44 or 45 or 46 or 47 or 48 or 48 or 49 or 50 or 51 or 52 or 53 or 54 or 55 or 56 or 57 or 58 or 59 or 60 or 61
64. 62 and 63
65. limit 64 to yr="2008 -Current"

COCHRANE searched 29/11/2022

IDSearchHits
#1MeSH descriptor: [Breast Feeding] explode all trees
#2MeSH descriptor: [Child Nutrition Sciences] explode all trees
#3MeSH descriptor: [Infant Nutritional Physiological Phenomena] explode all trees
#4MeSH descriptor: [Feeding Behavior] explode all trees
#5MeSH descriptor: [Infant Care] explode all trees
#6MeSH descriptor: [Perinatal Care] explode all trees
#7MeSH descriptor: [Postnatal Care] explode all trees
#8MeSH descriptor: [Infant Health] explode all trees
#9MeSH descriptor: [Mobile Applications] explode all trees
#10MeSH descriptor: [Smartphone] explode all trees
#11MeSH descriptor: [Cell Phone] explode all trees
#12MeSH descriptor: [Computers, Handheld] explode all trees
#13MeSH descriptor: [Telemedicine] explode all trees
#14MeSH descriptor: [Parenting] explode all trees
#15((breastfeeding OR breastfed OR “breast feeding” OR “breast fed” OR “perinatal health” OR “postnatal health” OR “infant health” OR “perinatal care” OR “postnatal care” OR “infant care” OR feeding OR “infant nutrition*” OR lactation OR lactating OR “breast milk” OR “child nutrition*” OR “feeding behavior” OR parenting)):ti,ab,kw37105
#16(“mobile phone” OR “cell phone” OR smartphone OR IOS OR iPhone OR Android OR iPad OR “handheld computer” OR “personal digital assistant” OR app OR apps OR “mobile app“ OR “mobile application“ OR “mobile apps” OR “mobile health” OR mHealth OR m-Health OR “electronic health” OR eHealth OR e-Health OR “digital health” OR telehealth OR telemedicine OR telelactation OR “mobile technolog*” OR “portable software” OR portable OR “portable electronic” OR “wearable device” OR wireless):ti,ab,kw (Word variations have been searched)
#17#1 OR #2 OR #3 OR #4 OR #5 OR #6 OR #7 OR #8 OR #14 OR #15
#18#9 OR #10 OR #11 OR #12 OR #13 OR #16
#19#17 AND #18 with Cochrane Library publication date between Jul 2008 and November 2022

ACM Digital Library searched 29/11/2022

1. [Abstract: breastfeeding]
2. [Abstract: breastfed]
3. [Abstract: "breast feeding"]
4. [Abstract: "breast fed"]
5. [Abstract: "perinatal health"]
6. [Abstract: "postnatal health"]
7. [Abstract: "infant health"]
8. [Abstract: "perinatal care"]
9. [Abstract: "postnatal care"]
10. [Abstract: "infant care"]
11. [Abstract: feeding]
12. [Abstract: "infant nutrition*"]
13. [Abstract: lactation]

14. [Abstract: lactating]
15. [Abstract: "breast milk"]
16. [Abstract: "child nutrition*"]
17. [Abstract: "feeding behavior"]
18. [Abstract: parenting]
19. [Abstract: "mobile phone*"]
20. [Abstract: "cell phone"]
21. [Abstract: smartphone]
22. [Abstract: ios]
23. [Abstract: iphone]
24. [Abstract: android]
25. [Abstract: ipad]
26. [Abstract: "handheld computer"]
27. [Abstract: "personal digital assistant"]
28. [Abstract: app]
29. [Abstract: apps]
30. [Abstract: "mobile app"]
31. [Abstract: "mobile apps"]
32. [Abstract: "mobile application"]
33. [Abstract: "mobile health"]
34. [Abstract: mhealth]
35. [Abstract: "m-health"]
36. [Abstract: "electronic health"]
37. [Abstract: ehealth]
38. [Abstract: e-health]
39. [Abstract: "digital health"]
40. [Abstract: telehealth]
41. [Abstract: telemedicine]
42. [Abstract: telelactation]
43. [Abstract: "mobile technolog*"]
44. [Abstract: "portable software"]
45. [Abstract: portable]
46. [Abstract: "portable electronic"]
47. [Abstract: "wearable device"]
48. [Abstract: wireless]
49. [Keywords: breastfeeding]
50. [Keywords: breastfed]
51. [Keywords: "breast feeding"]
52. [Keywords: "breast fed"]
53. [Keywords: "perinatal health"]
54. [Keywords: "postnatal health"]
55. [Keywords: "infant health"]
56. [Keywords: "perinatal care"]
57. [Keywords: "postnatal care"]
58. [Keywords: "infant care"]
59. [Keywords: feeding]
60. [Keywords: "infant nutrition*"]
61. [Keywords: lactation]
62. [Keywords: lactating]
63. [Keywords: "breast milk"]
64. [Keywords: "child nutrition*"]
65. [Keywords: "feeding behavior"]
66. [Keywords: parenting]
67. [Keywords: "mobile phone"]
68. [Keywords: "cell phone"]
69. [Keywords: smartphone]
70. [Keywords: ios]
71. [Keywords: iphone]
72. [Keywords: android]
73. [Keywords: ipad]
74. [Keywords: "handheld computer"]
75. [Keywords: "personal digital assistant"]

76. [Keywords: app]
77. [Keywords: apps]
78. [Keywords: "mobile app"]
79. [Keywords: "mobile application"]
80. [Keywords: "mobile apps"]
81. [Keywords: "mobile health"]
82. [Keywords: mhealth]
83. [Keywords: m-health]
84. [Keywords: "electronic health"]
85. [Keywords: ehealth]
86. [Keywords: e-health]
87. [Keywords: "digital health"]
88. [Keywords: telehealth]
89. [Keywords: telemedicine]
90. [Keywords: telelactation]
91. [Keywords: "mobile technolog*"]
92. [Keywords: "portable software"]
93. [Keywords: portable]
94. [Keywords: "portable electronic"]
95. [Keywords: "wearable device"]
96. [Keywords: wireless]
97. 1 or 2 or 3 or 4 or 5 or 6 or 7 or 8 or 9 or 10 or 11 or 12 or 13 or 14 or 15 or 16 or 17 or 18
98. 19 or 20 or 21 or 22 or 23 or 24 or 25 or 26 or 27 or 28 or 29 or 30 or 31 or 32 or 33 or 34 or 35 or 36 or 37 or 38 or 39 or 40 or 41 or 42 or 43 or 44 or 45 or 46 or 47 or 48 or 48
99. 97 and 98
100. 49 or 50 or 51 or 52 or 53 or 54 or 55 or 56 or 57 or 58 or 59 or 60 or 61 or 66
101. 67 or 68 or 69 or 70 or 71 or 72 or 73 or 74 or 75 or 76 or 77 or 78 or 79 or 80 or 81 or 82 or 83 or 84 or 85 or 86 or 87 or 88 or 89 or 90 or 91 or 92 or 93 or 94 or 95 or 96
102. 100 and 101
103. 99 and 102
104. 103 and [Publication Date: (01/01/2008 TO 11/29/2022)]

Appendix 2. Excluded studies and reasons for exclusion

Study ID	Reason for exclusion
1. Achouche V et al., 2022 (Achouche et al., 2022)	No relevant outcomes
2. Ahsan A et al., 2013 (Ahsan & Raihan, 2013)	Intervention not a mobile app
3. Akber S et al., 2019 (Akber et al., 2019)	Study design
4. Alam M et al., 2017 (Alam et al., 2017)	Intervention not a mobile app
5. Aloysius A et al., 2020 (Aloysius et al., 2020)	Study design
6. Banerjee J et al., 2020 (Banerjee et al., 2020)	Duplicate publication (see Aloysius et al.)
7. Bender W et al., 2022 (Bender et al., 2022)	Intervention not a mobile app
8. Chawla D et al., 2021 (Chawla et al., 2021)	Study design
9. Farr RS et al., 2019 (Farr et al., 2019)	Study design
10. Flax VL et al., 2022 (Flax et al., 2022)	Target app users
11. Griffin L et al., 2020 (Griffin et al., 2020)	Study design
12. Hägi-Pedersen, M. et. al. 2020 (Hägi-Pedersen et al., 2020)	Study design
13. Holm KG et al., 2019 (Holm et al., 2019)	Study design
14. Kapinos K et al, 2019 (Kapinos et al., 2019)	Study design
15. Lewkowitz AK et al., 2020 (Lewkowitz et al., 2020)	Study design
16. Ma T et al., 2022 (Ma et al., 2022)	Target app users
17. Maslowsky J et al., 2016 (Maslowsky et al., 2016)	Intervention not a mobile app
18. McGrath R et al., 2019 (McGrath et al., 2019)	Study design
19. Miremberg H et al., 2021 (Miremberg et al., 2021)	Intervention not a mobile app
20. Nguyet TT et al., 2021 (Nguyet et al., 2021)	Intervention not a mobile app
21. Ogaji DS et al., 2021 (Ogaji et al., 2021)	Intervention not a mobile app
22. Rahman F et al., 2019 (Rahman et al., 2019)	Duplicate publication (see Farr et al.)
23. Russel CG et al., 2018 (Russell et al., 2018)	Study design
24. Seguranyes G et al., 2014 (Seguranyes et al., 2014)	Intervention not a mobile app
25. Seyyedi N et al., 2021 (Seyyedi et al., 2021)	No relevant outcomes
26. Shorey S et al., 2018 (Shorey et al., 2019)	No relevant outcomes
27. Short V et al., 2021 (Short et al., 2021)	Target app users
28. Vartanian, K., et al. 2020 (Vartanian et al., 2020)	Report of included study
29. Ward VC et al., 22020 (Ward et al., 2020)	Intervention not a mobile app
30. Wheaton N et al., 2018 (Wheaton et al., 2018)	Study design
31. Wilson JL et al., 2012 (Wilson et al., 2012)	Intervention not a mobile app

Appendix 3. Assessment of the risk of bias in individual studies A. RCTs, B. quasi-experimental studies, C. cohort studies

A.

Domain assessed/Study ID	Borgen et al.	Bunik et al.	Doan et al.	Scott et al.	Uscher-Pines et al.	Wu et al.
1. Was true randomization used for assignment of participants to treatment groups?	YES	YES	YES	YES	YES	YES
2. Was allocation to treatment groups concealed?	YES	UNCLEAR	UNCLEAR	NO	YES	YES
3. Were treatment groups similar at the baseline?	NO	NO	YES	YES	NO	NO
4. Were participants blind to treatment assignment?	NO	NO	UNCLEAR	NO	NO	NO
5. Were those delivering treatment blind to treatment assignment?	UNCLEAR	UNCLEAR	UNCLEAR	UNCLEAR	UNCLEAR	UNCLEAR
6. Were outcomes assessors blind to treatment assignment?	NO	NO	UNCLEAR	NO	NO	NO
7. Were treatment groups treated identically other than the intervention of interest?	YES	YES	YES	YES	YES	YES
8. Was follow up complete and if not, were differences between groups in terms of their follow up adequately described and analyzed?	UNCLEAR	YES	YES	YES	YES	YES
9. Were participants analyzed in the groups to which they were randomized?	YES	YES	YES	YES	YES	YES
10. Were outcomes measured in the same way for treatment groups?	YES	YES	YES	YES	YES	YES
11. Were outcomes measured in a reliable way?	YES	YES	YES	YES	YES	YES
12. Was appropriate statistical analysis used?	YES*	YES	YES	YES	YES*	YES*
13. Was the trial design appropriate, and any deviations from the standard RCT design (individual randomization, parallel groups) accounted for in the conduct and analysis of the trial?	YES	YES	YES	YES	YES	YES

B.

Domain assessed/Study ID	Laws et al.
1. Is it clear in the study what is the 'cause 'and what is the 'effect '(i.e. there is no confusion about which variable comes first)?	YES
2. Were the participants included in any comparisons similar?	NO
3. Were the participants included in any comparisons receiving similar treatment/care, other than the exposure or intervention of interest?	YES
4. Was there a control group?	YES
5. Were there multiple measurements of the outcome both pre and post the intervention/exposure?	YES
6. Was follow up complete and if not, were differences between groups in terms of their follow up adequately described and analyzed?	YES
7. Were the outcomes of participants included in any comparisons measured in the same way?	YES
8. Were outcomes measured in a reliable way?	YES
9. Was appropriate statistical analysis used?	YES*

* sample size was not based on a statistical power calculation (a feasibility study)

C.

Domain assessed/Study ID	Cawley et al.	Deave et al.
1. Were the two groups similar and recruited from the same population?	NO	YES
2. Were the exposures measured similarly to assign people to both exposed and unexposed groups?	YES	YES
3. Was the exposure measured in a valid and reliable way?	YES	YES
4. Were confounding factors identified?	YES	YES
5. Were strategies to deal with confounding factors stated?	YES	YES
6. Were the groups/participants free of the outcome at the start of the study (or at the moment of exposure)?	YES	YES
7. Were the outcomes measured in a valid and reliable way?	YES	YES
8. Was the follow up time reported and sufficient to be long enough for outcomes to occur?	YES	YES
9. Was follow up complete, and if not, were the reasons to loss to follow up described and explored?	NA	NO
10. Were strategies to address incomplete follow up utilized?	NA	NO
11. Was appropriate statistical analysis used?	YES	YES

NA, not applicable

Appendix 4. Primary outcomes measures and results from individual studies

Study ID	Study design	Outcome measures	Results – BF outcomes<br>Intervention vs control group/ App users vs non-users
Borgen et al., 2019	RCT	Any BF in 1st wk	69,6% (78/112) vs 74,4% (90/121), P = 0,42
		Cessation of BF <1 m	7.7% (6/78) vs 2.2% (2/90); Still BF: 72 (64,3%) vs 88 (72.7%)
		Cessation of BF at 1-2 m	2.6% (2/72) vs 4.4.% (4/88); Still BF: 70 (62.5%) vs 84 (69.4%)
		BF at ≥3 m	62.5% (70/112) vs 69.4% (84/121); P = 0.21 (all time points) ITT
Bunik et al., 2022	RCT	Exclusive BF at 3 m	PP: 58% 95% CI (49%-67%) of 117/205 vs 54% 95% CI (42%-66%) of 59/110<br>ITT: 36% 95% CI (29 -43%) 117/310 vs 36% 95% CI (26-46 %) of 59/157
		Exclusive BF at 6 m	PP: 47% 95% CI (38%-56%) of 102/212 vs 40% 95% CI (29%-53%) of 46/109; P for interaction 0.79<br>ITT: 30% (23-37%) of 102/310 vs 26% (18-35%) of 46/157; P for interaction 0.54<br>For the ITT, all participants with a missing data re exclusive BF at 3 and 6 m assumed to be not BF exclusively
		Any BF at 3 m	PP: 76% 95% CI (67%-83%) of 148/205 vs 77% 95% CI (66%-86%) of 81/110<br>ITT: 148/310 vs 81/157
		Any BF 6 at m	PP: 66% 95% CI (57%-74%) of 137/212 vs 66% 95% CI (53%-77%) of 70/109; P for interaction 0.85;<br>ITT 137/310 vs 70/157
Cawley et al., 2020	Retrospective cohort study	BF ≥ 6 m	73,1% (118/162) vs 59,6% (226/379); P =.012; aOR = 1,75; Adjusted model controlled for age, race, household income, parity, insurance type)
Deave et al., 2019	Cohort study	Exclusive BF at 1 wk	57,0% (65/114) vs 51,1 % (91/182) χ^2 = 0.97, P = 0,325<br>OR = 1.27 (95% CI [0.79, 2.04]); aOR = 1.13 (95% CI [0.67, 1.90])
		Exclusive BF at 1 m	49,1% (55/114) vs 37,4% (67/182) χ^2 = 3.86, P = 0,05; OR = 1.61 (95% CI [1.00, 2.60]); aOR = 1.65 (95% CI [0.97, 2.80])
		Exclusive BF at 3 m	43,8% (49/114) vs 33,7% (61/182) χ^2 = 2.98, P = 0,084; OR = 1.53 (95% CI [0.94, 2.48]); aOR = 1.79 (95% CI [1.02, 3.16])
		Any BF at 1 wk	87,7% (100/114) vs 79,2% (141/182); χ^2 = 3.49, P = 0,062; OR = 1.87 (95% CI [0.96, 3.65]), aOR = 2.25 (95% CI [1.00, 5.06])
		Any BF at 1 m	84,8% (95/114) vs 67,6% (121/182) χ^2 = 10.68, P = 0,001; OR = 2.68 (95% CI [1.46, 4.90]); aOR = 3.08 (95% CI [1.49, 6.35])
		Any BF at 3 m	61,6% (69/114) vs 51,4% (93/182) χ^2 = 2.93, P = 0,087; OR = 1.52 (95% CI [0.94, 2.45]); aOR = 1.72 (95% CI [0.99, 2.99]); Adjusted model controlled for: IMD decile, education, technology use, use of any pregnancy/parenthood apps, baseline intention to BF
Doan et al., 2022	RCT	Early initiation of BF (within 2 h)	ITT: 29,6% (79/267) vs 22% (56/254), P < 0.05; OR = 1.52 (95% CI [1.02, 2.26]); aOR = 1.5 (95% CI [1.01, 2.24])
		Exclusive BF during hospital stay	21,5% (63/293) vs 14,5% (40/275), p < 0.05; OR = 1.62 (95% CI [1.04, 2.50]); aOR = 1.6 (95% CI [1.03, 2.48])
		Exclusive BF at 1 m	17,4% (51/293) vs 12,7% (35/275); OR = 1.45 (95% CI [0.91, 2.30]); aOR = 1.46 (95% CI [0.91, 2.34])
		Exclusive BF at 4 m	15,1% (44/292) vs 11,6% (32/275); OR = 1.35 (95% CI [0.83, 2.20]); aOR = 1.33 (95% CI [0.81, 2.12])
		Exclusive BF at 6 m	6,6% (19/289) vs 5,1% (14/274); OR = 1.31 (95% CI [0.64, 2.66]); aOR = 1.33 (95% CI [0.65, 2.72]); Adjusted model controlled for maternal age, education, prepregnancy BMI, parity, gestational age at enrolment, EBF intention

Laws et al., 2018	Quasi-Experimental study	Duration of any BF at 9 m	39.6 wks (95% CI [37.5, 41.8]) vs 39.0 wks (95% CI [37.3, 40.7]), P = 0.46
		Cessation of BF by 9 m	31.6% vs 28.5%; HR = 1.13 (95% CI [0.74, 1.74])
		Exclusive BF at 6 m	9% (10/111) vs 12.5% (20/160); aOR = 1.22 (95% CI [0.46, 3.32]); Adjusted model controlled for child and maternal age, gender, parity, dummy use, smoking status, country of birth, parental education and working status, maternal prepregnancy BMI, and house income
Scott et al., 2021	RCT	Exclusive BF at 6 wks	ITT: 69% (155/224) vs 71% (153/215); OR = 0.91 (95% CI [0.63, 1.30])
		Exclusive BF at 26 wks	ITT: 4% (7/184) vs 5% (9/184); OR = 0.82 (95% CI [0.37,1.80])
		Any BF at 6 wks	ITT: 93% (208/224) vs 94% (202/215); OR = 0.85 (95% CI [0.44, 1.61])
		Any BF at 26 wks	ITT: 78% (144/184) vs 80% (147/184); OR = 0.90 (95% CI [0.59, 1.37])<br>PP and analyses (PP, ITT) of the imputed dataset – similar/NS results
		Exclusive BF cessation <6 m	ITT: HR = 1.04 (95% CI [0.87,1.25]) PP/imputed – similar results
		Any BF cessation <6 m	ITT: HR = 1.08 (95% CI [0.73-1.58]) PP/imputed – similar results
Uscher-Pines et al., 2019	RCT	Exclusive BF at 12 wks	ITT: Among BF mothers: 51% (34/67) vs 46% (29/63), P = 0.47<br>Among total participants: 36% (34/94) vs 31% (29/93)<br>IV (instrumental variables) model: Among BF mothers: 56% vs. 45% , P = 0.48<br>the instrumental variable model applied to estimate the effect in the treated participants - only 50 % of the App group participated in ≥ 1 video call
		Any BF at 12 wks	ITT analysis: 71% (67/94) vs 68% (63/93), P = 0.73<br>IV (instrumental variables) model: 73% vs. 68%, P = 0.74
Wu et al., 2020	RCT	Early initiation of BF (within 1 h)	62.8% (93/148) vs 72.7% (101/139); OR = 0.64 (95% CI [0.39, 1.05]); aOR = 0.66 (95% CI [0.40, 1.09])
		Exclusive BF at 0-1 m (0-60 d)	81.1% (120/148) vs 63.3% (88/139); OR = 2.49 (95% CI [1.45, 4.25]); aOR = 2.75 (95% CI [1.58, 4.78])
		Exclusive BF at 2-3 m (61-120 d)	73% (111/152) vs 63,2% (96/152), OR = 1.47 (95% CI [0.91, 2.38]); aOR = 1.53 (95% CI [0.94, 2.49])
		Exclusive BF at 4-5 m (121-180 d)	46.3% (50/108) vs 42,2% (46/109), OR = 1.18 (95% CI [0.69, 2.02]); aOR = 1.37 (95% CI [0.78, 2.39])
		Predominant BF at 0-1 m (0-60 d)	83.8% (124/148) vs 67.6% (94/139); OR = 2.47 (95% CI [1.41, 4.34]); aOR = 2.77 (95% CI [1.55, 4.96])
		Predominant BF 2-3 m (61-120 d)	74.3% (113/152) vs 65.1% (99/152), OR = 1.55 (95% CI [0.95, 2.54]); aOR = 1.60 (95% CI [0.96, 2.64])
		Predominant BF 4-5 m (121-180 d)	53.7% (58/108) vs 48,6% (53/109), OR = 1.23 (95% CI [0.72, 2.09]); aOR = 1.46 (95% CI [0.82, 2.51]
		Any BF at 0-1 m (0-60 d)	98.6% (146/148) vs 95.7% (133/139); OR = 3.29 (95% CI [0.65, 16.59]); aOR = 3.09 (95% CI [0.61, 15.79])
		Any BF 2-3 m (61-120 d)	94.7% (144/152) vs 94,1% (143/152), OR = 1.13 (95% CI [0.43, 3.02]); aOR = 1.27 (95% CI [0.47, 3.46])
		Any BF 4-5 m (121-180 d)	93.5% (101/108) vs 89.0% (97/109), OR = 1.78 (95% CI [0.68, 4.72]); aOR = 2.34 (95% CI [0.84, 6.54])
		Ever BF (0-1 m)	147/148 (99.3%) vs 136/139 (97.8%); OR = 3.24 (95% CI [0.33, 31.54]); aOR = 3.42 (95% CI [0.34, 34.4]); Adjusted model controlled for baseline parity

aOR, adjusted odds ratio; BF, breastfeeding; CI, confidence interval; d, days; HR, hazard ratio; ITT, intention-to-treat analysis; m, months; NA, not available; OR, odds ratio; PP, per protocol analysis; RCT, randomized controlled trial; wk(s), week(s)

Appendix 5. Secondary outcomes - results

Study ID	Study design	App usage metrics and usability outcomes	Secondary outcome measures	Results
Borgen et al., 2019	RCT	NA	Adverse events	None reported
Bunik et al., 2022	RCT	>80% of mothers registered the App; mothers engaged with the content; the App perceived as useful and dependable (especially the texting feature); median IQR count of interactions with the App not significantly different between the study groups ($p = 0.44$). No significant difference in terms of BF outcomes between those who had vs those who did not have any engagement ($p = 0.85$); positive effect of engagement on BF (88% vs 72%) but no differential effect of engagement between study groups ($p = 0.90$)	BF Knowledge-Attitudes score (Iowa Infant Feeding Attitude Scale)	No differences between the groups
			BF self- efficacy/ confidence (BSES-LF)	Significantly higher in the intervention group ($p<0.05$)
			Perceived BF Support Scale	
			Adverse events	None reported
Cawley et al., 2020	Retrospective cohort study	NA	NA	NA
Deave et al., 2019	Cohort study	Participants engaged more in the passive element of the App (based on a subgroup analysis, n=51); no significant association between primary outcomes (parenting self-efficacy, mental well-being) and usage of passive and active App elements or the level of usage high vs low users or high vs non-users	NA	NA
Doan et al., 2022	RCT	NA	NA	NA
Laws et al., 2018	Quasi-Experimental study	86.4% opted to access the program via the App, and 13.6% via the website/SMS. 74.8% of App participants downloaded the app; 8.0% of all notifications opened during the study. App usage declined over time (92.0% used the app at least once on enrolment, 38.2% at study completion at offspring age of 8-9 m); similarly the usage frequency declined over time; overall high level of satisfaction with the program; technical problems experienced >25% of parents	NA	NA
Scott et al., 2021	RCT	80.4% participants in the intervention group downloaded the App; the level of engagement not associated with BF outcomes	Maternal BF self-efficacy (BSES- SF)	No significant differences between the study groups-
			Partner postpartum support (PPSS)	
Uscher-Pines et al., 2019	RCT	Only 50% of the intervention group participated in ≥ 1 video call (among those 51% in 1, 15% in 2, and 34% in 3+ video calls; 91% of the participants satisfied with the advice received	Satisfaction with the BF experience	Less likely to be reported in the intervention group (the differences not statistically significant)
Wu et al., 2020	RCT	A total of 108 messages published in the module and read > 8892 times; The top 5 read messages: benefits of BF (393 times); importance of the early BF initiation (284 times); BF positions and latching-on (242 times); what is early initiation (214 times); WHO recommendation: children should be exclusively BF from birth to 6 m (214 times)	Mothers' knowledge on BF practices	All knowledge indicators low at baseline, with improvement at follow-up; no differences between the study groups at all time points

App, application; BF, breastfeeding; Breastfeeding Self-Efficacy Scale – Short Form/Long Form, BSES-SF/LF; d, days; IQR, interquartile range; m, months; NA, not available/applicable; Postpartum Partner Support Scale, PPSS; RCT, randomized controlled trial; wk(s), week(s);

REFERENCES:

Achouche, V., Piollet, M., Temame, M., Cao, X. N., D'Herbemont, V., Moreau, J., & Wendland, J. (2022). The impact of a mobile application on parental attitudes, their knowledge of child development, and sense of parenting self-competence: A pilot study. *Annales Medico-Psychologiques*. https://doi.org/https://dx.doi.org/10.1016/j.amp.2022.09.010 (Impact d'une application mobile sur les attitudes des parents, leurs connaissances du developpement de l'enfant et leur sentiment de competence parentale : une etude pilote)

Ahsan, A., & Raihan, A. (2013). *Understanding mHealth impact among Aponjon (MAMA Bangladesh) subscribers through a phone survey in Bangladesh* Proceedings of the Sixth International Conference on Information and Communications Technologies and Development: Notes - Volume 2, Cape Town, South Africa. https://doi.org/10.1145/2517899.2517920

Akber, S., Mahmood, H., Abbasi, S., Mahmood, H., Hazir, T., Fatima, R., Yaqoob, A., Wali, A., Alam, A., Dibley, M. J., Sheraz, S. Y., & Najmi, H. (2019). Effectiveness of a mobile health intervention on infant and young child feeding among children <= 24 months of age in rural Islamabad over six months duration. *F1000Research, 8*, 551. https://doi.org/http://dx.doi.org/10.12688/f1000research.17037.3

Alam, M., D'Este, C., Banwell, C., & Lokuge, K. (2017). The impact of mobile phone based messages on maternal and child healthcare behaviour: a retrospective cross-sectional survey in Bangladesh. *BMC health services research, 17*(1), 434. https://doi.org/http://dx.doi.org/10.1186/s12913-017-2361-6

Aloysius, A., Mitchell, K., Silva, I., Godambe, S. V., Deierl, A., Banerjee, J., & Rallis, D. (2020). Improving infant outcomes through implementation of a family integrated care bundle including a parent supporting mobile application. *Archives of Disease in Childhood: Fetal and Neonatal Edition, 105*(2), F172-F177. https://doi.org/http://dx.doi.org/10.1136/archdischild-2018-316435

Banerjee, J., Aloysius, A., Mitchell, K., Silva, I., Rallis, D., Godambe, S. V., & Deierl, A. (2020, Mar). Improving infant outcomes through implementation of a family integrated care bundle including a parent supporting mobile application. *Arch Dis Child Fetal Neonatal Ed, 105*(2), 172-177. https://doi.org/10.1136/archdischild-2018-316435

Bender, W., Levine, L., & Durnwald, C. (2022, Nov 1). Text Message-Based Breastfeeding Support Compared With Usual Care: A Randomized Controlled Trial. *Obstet Gynecol, 140*(5), 853-860. https://doi.org/10.1097/aog.0000000000004961

Borgen, I., Småstuen, M. C., Jacobsen, A. F., Garnweidner-Holme, L. M., Fayyad, S., Noll, J., & Lukasse, M. (2019, Nov 11). Effect of the Pregnant+ smartphone application in women with gestational diabetes mellitus: a randomised controlled trial in Norway. *BMJ Open, 9*(11), e030884. https://doi.org/10.1136/bmjopen-2019-030884

Bunik, M., Jimenez-Zambrano, A., Solano, M., Beaty, B. L., Juarez-Colunga, E., Zhang, X., Moore, S. L., Bull, S., & Leiferman, J. A. (2022). Mother's Milk MessagingTM: trial evaluation of app and texting for breastfeeding support. *BMC Pregnancy and Childbirth, 22*(1), 660. https://doi.org/https://dx.doi.org/10.1186/s12884-022-04976-6

Cawley, C., Buckenmeyer, H., Jellison, T., Rinaldi, J. B., & Vartanian, K. B. (2020). Effect of a Health System-Sponsored Mobile App on Perinatal Health Behaviors: Retrospective Cohort Study. *JMIR mhealth and uhealth, 8*(7), e17183. https://doi.org/http://dx.doi.org/10.2196/17183

Chawla, D., Thukral, A., Deorari, A., & Kumar, P. (2021). Harnessing mobile technology to deliver evidence-based maternal-infant care. *Seminars in Fetal and Neonatal Medicine, 26*(1), 101206. https://doi.org/http://dx.doi.org/10.1016/j.siny.2021.101206

Deave, T., Ginja, S., Goodenough, T., Bailey, E., Piwek, L., Coad, J., Day, C., Nightingale, S., Kendall, S., & Lingam, R. (2019). The Bumps and BaBies Longitudinal Study (BaBBLeS): a multi-site cohort study of first-time mothers to evaluate the effectiveness of the Baby Buddy app. *Mhealth, 5*, 42. https://doi.org/10.21037/mhealth.2019.08.05

Doan, T. T. D., Pham, N. M., Binns, C., Lee, A., Zhao, Y., Dinh, T. P. H., & Bui, T. T. H. (2022, Oct). Effect of a Mobile Application on Breastfeeding Rates Among Mothers Who Have Cesarean Deliveries: A Randomized Controlled Trial. *Breastfeed Med, 17*(10), 832-840. https://doi.org/10.1089/bfm.2022.0088

Farr, R. S., Rahman, F., O'Riordan, M. A., & Furman, L. (2019, Dec). Assessing the Feasibility and Effectiveness of Two Prenatal Breastfeeding Intervention Apps in Promoting Postpartum In-Hospital Exclusive Breastfeeding. *Breastfeed Med, 14*(10), 724-730. https://doi.org/10.1089/bfm.2019.0053

Flax, V. L., Ipadeola, A., Schnefke, C. H., Ralph-Opara, U., Adeola, O., Edwards, S., Bose, S., & Brower, A. O. (2022, May 5). Breastfeeding Interpersonal Communication, Mobile Phone Support, and Mass Media Messaging Increase Exclusive Breastfeeding at 6 and 24 Weeks Among Clients of Private Health Facilities in Lagos, Nigeria. *J Nutr, 152*(5), 1316-1326. https://doi.org/10.1093/jn/nxab450

Griffin, L., Lewkowitz, A., Lopez, J., Macones, G., & Cahill, A. (2020). Does increased use of breastfeeding smartphone applications improve breastfeeding rates among low-income women? *Breastfeeding medicine*, *15*(10), A28-A29. https://doi.org/http://dx.doi.org/10.1089/bfm.2020.29162.abstracts (25th Annual International Meeting of the Academy of Breastfeeding Medicine. Online.)

Hägi-Pedersen, M. B., Dessau, R. B., Norlyk, A., Stanchev, H., & Kronborg, H. (2020, Mar 30). Comparison of video and in-hospital consultations during early in-home care for premature infants and their families: A randomised trial. *J Telemed Telecare*, 1357633x20913411. https://doi.org/10.1177/1357633x20913411

Holm, K. G., Clemensen, J., Brødsgaard, A., Smith, A. C., Maastrup, R., & Zachariassen, G. (2019). Growth and breastfeeding of preterm infants receiving neonatal tele-homecare compared to hospital-based care. *J Neonatal Perinatal Med, 12*(3), 277-284. https://doi.org/10.3233/npm-18143

Kapinos, K., Kotzias, V., Bogen, D., Ray, K., Demirci, J., Rigas, M. A., & Uscher-Pines, L. (2019, Sep 3). The Use of and Experiences With Telelactation Among Rural Breastfeeding Mothers: Secondary Analysis of a Randomized Controlled Trial. *J Med Internet Res, 21*(9), e13967. https://doi.org/10.2196/13967

Laws, R. A., Denney-Wilson, E. A., Taki, S., Russell, C. G., Zheng, M., Litterbach, E. K., Ong, K. L., Lymer, S. J., Elliott, R., & Campbell, K. J. (2018, Apr 19). Key Lessons and Impact of the Growing Healthy mHealth Program on Milk Feeding, Timing of Introduction of Solids, and Infant Growth: Quasi-Experimental Study. *JMIR Mhealth Uhealth*, *6*(4), e78. https://doi.org/10.2196/mhealth.9040

Lewkowitz, A. K., Lopez, J. D., Werner, E. F., Ranney, M. L., Macones, G. A., Rouse, D. J., Savitz, D. A., & Cahill, A. G. (2020). Effect of a Novel Smartphone Application on Breastfeeding Rates Among Low-Income, First-Time Mothers Intending to Exclusively Breastfeed: secondary Analysis of a Randomized Controlled Trial [Journal: Article in Press]. *Breastfeeding medicine*. https://doi.org/10.1089/bfm.2020.0240

Ma, T., Chang, K., Alyusuf, A., Bajracharya, E., Washio, Y., Kelly, P. J., Bellad, R. M., Mahantashetti, N. S., Charantimath, U., Short, V. L., Lalakia, P., Jaeger, F., Goudar, S., & Derman, R. (2022, Sep 8). Design, Development, and Testing of BEST4Baby, an mHealth Technology to Support Exclusive Breastfeeding in India: Pilot Study. *JMIR Form Res, 6*(9), e32795. https://doi.org/10.2196/32795

Maslowsky, J., Frost, S., Hendrick, C. E., Trujillo Cruz, F. O., & Merajver, S. D. (2016, Jul). Effects of postpartum mobile phone-based education on maternal and infant health in Ecuador. *Int J Gynaecol Obstet, 134*(1), 93-98. https://doi.org/10.1016/j.ijgo.2015.12.008

McGrath, R., Caruth, G., Miletin, J., Doolan, A., Barrett, T., O'Cuiv, L., Walsh, A., Biesma, R., & Turner, M. (2019). 'Mycoombe' mhealth app - A pilot study. *Archives of disease in childhood, 104*(Supplement 3), A136. https://doi.org/http://dx.doi.org/10.1136/archdischild-2019-epa.314 (9th Europaediatrics Congress of Royal College of Paediatrics and Child Health. Dublin Ireland.)

Miremberg, H., Yirmiya, K., Rona, S., Gonen, N., Kovo, M., Bar, J., Weiner, E., Marom, O., & Pohol, A. (2021). 98 Smartphone-based counseling and support platform and the effect on postpartum lactation-a randomized controlled trial. *American journal of obstetrics and gynecology, 224*(2 Supplement), S69. https://doi.org/http://dx.doi.org/10.1016/j.ajog.2020.12.101 (SMFM 41st Annual Meeting: The Pregnancy Meeting. Virtual, Online.)

Nguyet, T. T., Huy, N. V. Q., & Kim, Y. (2021, Dec 31). Effects of a newborn care education program using ubiquitous learning on exclusive breastfeeding and maternal role confidence of first-time mothers in Vietnam: a quasi-experimental study. *Korean J Women Health Nurs, 27*(4), 278-285. https://doi.org/10.4069/kjwhn.2021.12.03

Ogaji, D. S., Arthur, A. O., & George, I. (2021). Effectiveness of Mobile Phone-Based Support on Exclusive Breastfeeding and Infant Growth in Nigeria: A Randomized Controlled Trial. *Journal of tropical pediatrics, 67*(1), fmaa076. https://doi.org/http://dx.doi.org/10.1093/tropej/fmaa076

Rahman, F., Farr, R. S., O'Riordan, M. A., & Furman, L. (2019). Assessing the Feasibility and Effectiveness of Two Prenatal Breastfeeding Intervention Apps in Promoting Postpartum In-Hospital Exclusive Breastfeeding. *Breastfeeding medicine, 14*(10), 724-730. https://doi.org/http://dx.doi.org/10.1089/bfm.2019.0053

Russell, C. G., Denney-Wilson, E., Laws, R. A., Abbott, G., Zheng, M., Lymer, S. J., Taki, S., Litterbach, E. V., Ong, K. L., & Campbell, K. J. (2018, Apr 25). Impact of the Growing Healthy mHealth Program on Maternal Feeding Practices, Infant Food Preferences, and Satiety Responsiveness: Quasi-Experimental Study. *JMIR Mhealth Uhealth, 6*(4), e77. https://doi.org/10.2196/mhealth.9303

Scott, J. A., Burns, S. K., Hauck, Y. L., Giglia, R. C., Jorgensen, A. M., White, B. K., Martin, A., Robinson, S., Dhaliwal, S. S., Binns, C. W., & Maycock, B. R. (2021, Apr 12). Impact of a Face-To-Face Versus Smartphone App Versus Combined Breastfeeding Intervention

Targeting Fathers: Randomized Controlled Trial. *JMIR Pediatr Parent*, *4*(2), e24579. https://doi.org/10.2196/24579

Seguranyes, G., Costa, D., Fuentelsaz-Gallego, C., Beneit, J. V., Carabantes, D., Gómez-Moreno, C., Palacio-Tauste, A., Pauli, A., & Abella, M. (2014, Jun). Efficacy of a videoconferencing intervention compared with standard postnatal care at primary care health centres in Catalonia. *Midwifery, 30*(6), 764-771. https://doi.org/10.1016/j.midw.2013.08.004

Seyyedi, N., Rahmatnezhad, L., Mesgarzadeh, M., Khalkhali, H., Seyyedi, N., & Rahimi, B. (2021, Sep 20). Effectiveness of a smartphone-based educational intervention to improve breastfeeding. *Int Breastfeed J*, *16*(1), 70. https://doi.org/10.1186/s13006-021-00417-w

Shorey, S., Ng, Y. P. M., Ng, E. D., Siew, A. L., Mörelius, E., Yoong, J., & Gandhi, M. (2019, Feb 13). Effectiveness of a Technology-Based Supportive Educational Parenting Program on Parental Outcomes (Part 1): Randomized Controlled Trial. *J Med Internet Res, 21*(2), e10816. https://doi.org/10.2196/10816

Short, V. L., Bellad, R. M., Kelly, P. J., Washio, Y., Ma, T., Chang, K., Majantashetti, N. S., Charantimath, U. S., Jaeger, F. J., Lalakia, P., Goudar, S. S., & Derman, R. (2021, Jan 17). Feasibility, acceptability, and preliminary impact of an mHealth supported breastfeeding peer counselor intervention in rural India. *Int J Gynaecol Obstet*. https://doi.org/10.1002/ijgo.13599

Uscher-Pines, L., Ghosh-Dastidar, B., Bogen, D. L., Ray, K. N., Demirci, J. R., Mehrotra, A., & Kapinos, K. A. (2019). Feasibility and Effectiveness of Telelactation Among Rural Breastfeeding Women [Journal: Article in Press]. *Academic pediatrics*. https://doi.org/10.1016/j.acap.2019.10.008

Vartanian, K., Rinaldi, J. B., Pieper, K., & Jellison, T. (2020). Effect of a health system sponsored mobile app on perinatal health behaviors. *Obstetrics and Gynecology, 135*(Supplement 1), 52S-53S. http://ovidsp.ovid.com/ovidweb.cgi?T=JS&PAGE=reference&D=emexa&NEWS=N&AN=633633972 (2020 American College of Obstetricians and Gynecologists Annual Meeting, ACOG 2020. Seattle, WA United States.)

Ward, V. C., Raheel, H., Abdalla, S., Pepper, K. T., Weng, Y., Kaimal, R., Bentley, J., Mehta, K. M., Dutt, P., Mitra, R., Sastry, P., Chamberlain, S., Godfrey, A., Shannon, M., Carmichael, S. L., Darmstadt, G. L., Mahapatra, T., Srikantiah, S., Borkum, E., Rangarajan, A., Sridharan, S., Rotz, D., Nanda, P., Tarigopula, U. K., Atmavilas, Y., & Bhattacharya, D. (2020). Impact of mHealth interventions for reproductive, maternal, newborn and child health and nutrition

at scale: BBC Media Action and the Ananya program in Bihar, India. *Journal of global health*, *10*(2), 021005. https://doi.org/http://dx.doi.org/10.7189/jogh.10.021005

Wheaton, N., Lenehan, J., & Amir, L. H. (2018, Nov). Evaluation of a Breastfeeding App in Rural Australia: Prospective Cohort Study. *J Hum Lact*, *34*(4), 711-720. https://doi.org/10.1177/0890334418794181

Wilson, J. L., Mallan, K. M., Daniels, L. A., Perry, R., Magarey, A., Mihrshahi, S., & Nicholson, J. M. (2012). Recruiting and engaging new mothers in nutrition research studies: lessons from the Australian NOURISH randomised controlled trial. *International journal of behavioral nutrition and physical activity*, *9*, 129. https://doi.org/http://dx.doi.org/10.1186/1479-5868-9-129

Wu, Q., Huang, Y., Liao, Z., van Velthoven, M. H., Wang, W., & Zhang, Y. (2020, Dec 3). Effectiveness of WeChat for Improving Exclusive Breastfeeding in Huzhu County China: Randomized Controlled Trial. *J Med Internet Res*, *22*(12), e23273. https://doi.org/10.2196/23273
